# Clinical pattern of antibiotic overuse and misuse in primary healthcare hospitals in the southwest of China

**DOI:** 10.1371/journal.pone.0214779

**Published:** 2019-06-26

**Authors:** Yue Chang, Sarunyou Chusri, Rassamee Sangthong, Edward McNeil, Jiaqi Hu, Wei Du, Duan Li, Xingying Fan, Hanni Zhou, Virasakdi Chongsuvivatwong, Lei Tang

**Affiliations:** 1 School of Medicine and Health Management, Guizhou Medical University, Guizhou, China; 2 Epidemiology Unit, Faculty of Medicine, Prince of Songkla University, Hat Yai, Songkhla, Thailand; 3 Division of Infectious Disease, Department of Internal Medicine, Prince of Songkla University, Hat Yai, Songkhla, Thailand; 4 Department of Biomedical Sciences, Prince of Songkla University, Hat Yai, Songkhla, Thailand; University of Campania, ITALY

## Abstract

**Purpose:**

Overuse and misuse of antibiotics are the primary risk factors for antibiotics resistance. Inadequate professional competence of primary care physicians might exacerbate these problems in China. This retrospective study aims to document the clinical pattern of antibiotics use and its overuse and misuse rates in rural primary care institutions and to evaluate the association between antibiotics use and characteristics of physicians and their patients.

**Methods:**

Medical records from 16 primary care hospitals in rural areas of Guizhou province, China were obtained from the Health Information System in 2018. Classification of unnecessary use, incorrect spectrum of antibiotic, escalated use of extended spectrum and combined antibiotics use was based on the Guiding Principle of Clinical Use of Antibiotics (2015, China) and guidelines from the US Centers for Disease Control and Prevention. Generalized Estimating Equations were employed to determine predictive factors for inappropriate antibiotics use.

**Results:**

A total of 74,648 antibiotics prescriptions were retrieved. Uncomplicated respiratory infection was the most common disease accounting for 58.6% of all prescriptions. The main antibiotic group used was penicillins (51.5%) followed by cephalosporins and macrolides (14% each). Of 57,009 patient visits, only 8.7% of the antibiotic prescriptions were appropriate. Combined use, escalated use of extended spectrum antibiotics, incorrect spectrum and unnecessary antibiotics use was found in 7.8%, 1.9%, 4.3% and 77.3% of patient visits, respectively, of which 28.7% were given intravenously. Antibiotics misuse was significantly more likely among newly employed physicians with lower levels of professional education. Adult patients and those who had public insurance had a higher risk of being prescribed unnecessary antibiotics.

**Conclusion:**

Overuse of antibiotics for uncomplicated respiratory infection and use of cephalosporins, macrolides and injection antibiotics in primary care are the major problems of clinical practice in rural areas of Guizhou.

## Introduction

Antibiotics resistance is a growing global public health issue [1]. Between 2000 and 2010 global antibiotics consumption grew by more than 50% based on data from 71 countries, including China [2]. Excessive consumption of antibiotics mainly leads to the development of antibiotics resistance. China consumes the second largest amount of antibiotics in the world [3, 4] with a prescription rate twice that recommended by the World Health Organization (WHO) [5] and rural areas have higher rates than urban areas [6]. In this regard, the Chinese government has introduced a number of regulations in the last decade, but it has not played a significant role in rural areas [7].

The majority of people in southwest China live in rural areas where primary care physicians usually provide the health services [8]. Most physicians there have non-degree training yet are allowed to prescribe antibiotics in the national list due to personnel shortages. Previous studies reported a high irrational antibiotics prescription use among primary care physicians [7, 9]. In addition, studies performed in Italy and Germany exploring the patterns of antibiotic prescriptions among primary care physicians found that the rate of inappropriate antibiotic use was high [10–12]. Strengthening the knowledge and practice of rational use of antibiotics among primary care physicians is one way to reduce antibiotic resistance. Thus, we need to understand how antibiotics are unnecessarily used, for example prescribed with incorrect choices for particular diseases, escalated (for example prescribing more expensive and broad spectrum antibiotics when cheaper and more specific antibiotics can give the same result) and used intravenously.

The aim of this study was to document the clinical patterns of antibiotics prescriptions in a rural primary care setting where physicians are mostly non-degree trained. The secondary objective was to determine the association between antibiotics use and various characteristics of patients and physicians.

## Materials and methods

Ethical approval: This research was approved by the Institutional Review Board of Prince of Songkhla University, Thailand (REC: 60-285-18-5). Ethics committees approved the consent procedure. All participants provided their written informed consent to participate in this study.

### Study setting

Out of 1,399 township public hospitals in Guizhou rural areas, 132 use the same health information system (HIS) developed by the Department of Public Health. Those that had more than three outpatient physicians were eligible for the study. Out of the 84 eligible hospitals, we randomly selected 31 to participate in the study.

### Data retrieval process

Only data from outpatient units were retrieved. Demographic characteristics of the patients, and education and work experiences of the physicians, were obtained from the Personnel Management Department. All patients prescribed antibiotics during February to August, 2018 were included in the analysis. The primary diagnoses of all diseases except for tuberculosis (since the treatment options are fixed and standardized), were grouped into 10 diagnostic categories according to the international classification for diseases version 10 (ICD-10) code [13]. Antibiotics were classified, based on the 2018 National Catalogue of Clinical Application of Antibacterial Drugs, into seven groups: penicillins, cephalosporins, macrolides, quinolones, lincosamides, nitroimidazoles, and aminoglycosides [14]. We focused on systemic antimicrobials excluding topical antimicrobial prescriptions such as ophthalmic ointments and skin creams.

### Categorization of appropriateness of antibiotic use

We used the National Health Commission of China for Guiding Principle of Clinical Use of Antibiotics introduced in 2015 (summarized in S1 File), and antibiotic use guidelines from the US Centers for Disease Control and Prevention (CDC) [15]. Appropriate antibiotic use in primary care settings was classified based on the diagnosis of probable bacterial infection. We categorised antibiotic use into appropriate use and inappropriate use. Inappropriate use was further categorised into four groups: 1) unnecessary use, e.g. prescribing antibiotics for viral infections, 2) incorrect spectrum of antibiotic, e.g. prescribing aminoglycoside for gram positive bacteria, 3) escalated use of extended spectrum antibiotics, e.g. prescribing cephalosporin instead of penicillin, and 4) combined use of antibiotics, defined as use of more than one antibiotic group per patient visit without any indications. In some cases, definitive bacterial culture penicillin (e.g. for acute pharyngitis/tonsillitis) is not available a presumptive narrow spectrum antibiotic, e.g. penicillin was considered as appropriate. Misuse of antibiotics was defined as unnecessary antibiotic use while overuse was defined as any other category of inappropriate antibiotic use.

### Data analysis

The units of analysis were both antibiotic prescriptions and patient visits; one physician may prescribe one or more antibiotics to a patient on a given day, but a patient can only visit one doctor per day. Cross-tabulation between the groups of antibiotics and the disease categories by ICD-10 diagnosis code was used to determine the pattern and appropriateness of antibiotics use. Misuse of antibiotic prescriptions was aggregated by antibiotic group. Bivariate cross-tabulations were initially used to identify potential risk factors of inappropriate antibiotic use. In order to account for the correlation of antibiotic prescriptions by the same physician, Generalized Estimating Equations (GEE) were used to determine independent predictors of antibiotic misuse controlling for possible confounding effects by other variables. All p-values were two-sided. R version 3.3.1 was used for all data management and analysis for the database (S1 Table).

## Results

A total of 96,509 antibiotic prescriptions among 57,009 patient visits were retrieved from the electronic database during the study period. The ten most common diagnoses among these patients accounted for over 77% of all prescriptions; therefore, we focused on only these ten diagnostic categories in the analysis.

Table 1 shows a comparison of the number of antibiotics prescribed for various common diseases stratified by appropriateness of use. Diseases of the respiratory system accounted for about 70% of all antibiotic prescriptions followed by diseases of the digestive system (13.1%) and genitourinary system (5.6%). Inappropriate use of antibiotics was found in 91.8% of the 74,648 prescriptions. The highest rate of inappropriate use was seen for symptoms, signs and abnormal clinical and laboratory finding not elsewhere classified (100%), diseases of the eye and adnexa (99%), and diseases of the skin and subcutaneous tissue (97.4%). Escalated use of extended spectrum antibiotics was common for patients with acute appendicitis (40.8%) and pneumonia, organism unspecified (31.3%). Incorrect spectrum of antibiotic was used frequently among patients with cystitis (45.8%) and other disorders of the urinary system (44.1%) whereas unnecessary use was common to all diseases. The highest rate of appropriate use was found in diseases of pulp and periapical tissues (71.2%), acute pharyngitis (69.7%), and arthritis (67.6%).

**Table 1:** Distribution of antibiotic prescriptions stratified by clinical diagnosis and appropriateness of use.

Diagnosis	Total	Appropriate use, n (%)	Inappropriate use, n (%)		
			Escalated use of extended spectrum	Incorrect spectrum	Unnecessary use
**1. Diseases of the respiratory system**	**51,987**	**4,248 (8.2)**	**126 (0.2)**	**1329 (2.6)**	**46,284 (89.0)**
*Upper respiratory tract infection*					
Acute tonsillitis	4,423	2,193 (49.6)	0 (0)	859 (19.4)	1,371 (31.0)
Acute pharyngitis	1,369	954 (69.7)	0 (0)	0 (0)	415 (30.3)
Acute sinusitis	332	81 (24.4)	11 (3.3)	56 (16.9)	184 (55.4)
Others	34,280	0 (0)	0 (0)	0 (0)	34,280 (100)
*Lower respiratory tract infection*					
Bronchitis, not specified as acute or chronic	1,725	887 (51.4)	0 (0)	333 (19.3)	505 (29.3)
Pneumonia, organism unspecified	367	133 (36.2)	115 (31.3)	81 (22.1)	38 (10.4)
Others	9,491	0 (0)	0 (0)	0 (0)	9,491 (100)
**2. Diseases of the digestive system**	**9,795**	**987 (10.1)**	**822 (8.4)**	**1,469 (15.0)**	**6,517 (66.5)**
Gingivitis and periodontal diseases	1,776	622 (35.0)	138 (7.8)	0 (0)	1,016 (52.2)
Diseases of pulp and periapical tissues	118	84 (71.2)	0 (0)	4 (3.4)	30 (25.4)
Gastritis and duodenitis	4,405	0 (0)	323 (7.3)	650 (14.8)	3,432 (77.9)
Cholecystitis	511	273 (53.4)	89 (17.4)	82 (16)	67 (13.1)
Acute appendicitis	49	8 (16.3)	20 (40.8)	0 (0)	21 (42.9)
Other non-infective gastroenteritis and colitis	1,755	0 (0)	252 (14.4)	733 (41.8)	770 (43.9)
Others	1,181	0 (0)	0 (0)	0 (0)	1,181 (100)
**3. Diseases of the genitourinary system**	**4,191**	**365 (8.7)**	**537 (32.5)**	**768 (18.3)**	**2,521 (60.2)**
Other female pelvic inflammatory disease	1367	0 (0)	273 (20)	294 (21.5)	800 (58.5)
Other inflammation of vagina and vulva	455	284 (62.4)	0 (0)	0 (0)	171 (37.6)
Cystitis	166	0 (0)	27 (16.3)	76 (45.8)	63 (38.0)
Urethritis and urethral syndrome	383	0 (0)	99 (25.8)	163 (42.6)	121 (31.6)
Other disorders of urinary system	533	81 (15.2)	138 (25.9)	235 (44.1)	79 (14.8)
Others	1287	0 (0)	0 (0)	0 (0)	1,287 (100)
**4. Diseases of the musculoskeletal system and connective tissue**	**2,318**	**669 (28.9)**	**77 (3.3)**	**22 (0.9)**	**1,550 (66.9)**
Other arthritis	989	669 (67.6)	77 (7.8)	22 (2.2)	221 (20.9)
Others	1329	0 (0)	0 (0)	0 (0)	1,329 (100)
**5. Injury, poisoning and certain other consequences of external causes**	**936**	**86 (9.2)**	**11 (1.2)**	**7 (0.7)**	**832 (88.9)**
Other and unspecified injuries of abdomen, lower back and pelvis	180	86 (47.8)	11 (6.1)	7 (3.9)	76 (42.2)
Others	756	0 (0)	0 (0)	0 (0)	756 (100)
**6. Diseases of the eye and adnexa**	**1,786**	**18 (1.0)**	**0 (0)**	**0 (0)**	**1,768 (99.0)**
Hordeolum and chalazion	141	18 (12.8)	0 (0)	0 (0)	123 (87.2)
Others	1645	0 (0)	0 (0)	0 (0)	1,645 (100)
**7. Diseases of the skin and subcutaneous tissue**	**965**	**25 (2.6)**	**14 (1.5)**	**9 (0.9)**	**917 (95.0)**
Cellulitis	65	25 (38.5)	14 (21.5)	9 (13.8)	17 (26.2)
Others	900	0 (0)	0 (0)	0 (0)	900 (100)
**8. Diseases of the circulatory system**	**952**	**103 (10.8)**	**0 (0)**	**0 (0)**	**849 (89.2)**
Rheumatic fever without mention of heart involvement	174	103 (59.2)	0 (0)	0 (0)	71 (40.8)
Others	778	0 (0)	0 (0)	0 (0)	778 (100)
**9. Certain infectious and parasitic diseases**	**627**	**66 (10.5)**	**30 (4.8)**	**80 (12.8)**	**451 (71.9)**
Other gastroenteritis and colitis of infectious and unspecified origin	246	66 (26.8)	30 (12.2)	80 (32.5)	70 (28.5)
Others	381	0 (0)	0 (0)	0 (0)	381 (100)
**10. Symptoms, signs and abnormal clinical and laboratory finding not elsewhere classified**	**1,091**	**0 (0)**	**0 (0)**	**0 (0)**	**1,091 (100)**
Abdominal and pelvic pain + fever of unknown origin + cough + pain in throat and chest + headache	1,091	0 (0)	0 (0)	0 (0)	1,091 (100)
**Total prescriptions**	**74,648**	**6,567 (8.8)**	**1,617 (2.2)**	**3,684 (4.9)**	**62,780 (84.1)**

Table 2 summarizes appropriateness of antibiotic use by group. Over a half (51.5%) of the prescribed antibiotics were penicillins. However, 84.4% of penicillins unnecessarily prescribed. The percentage of cephalosporins and macrolides prescribed was about 14% each, of which about 84.7% were unnecessarily prescribed. Quinolones, which should be used mainly as second-line antibiotics, was found in around 6% of all prescriptions, of which 46.2% were incorrect spectrum of antibiotic. Lincosamides, nitroimidazoles, and aminoglycosides were not commonly seen, however almost all were prescribed inappropriately.

**Table 2:** Distribution of antibiotic prescriptions stratified by antibiotic group and appropriateness of use.

Antibiotic group	Total	Appropriate use, n(%)	Inappropriate use, n (%)		
			Escalated use of extended spectrum	Incorrect spectrum	Unnecessary use
**Penicillins**	38,462 (51.5)	5,981 (15.6)	0 (0)	0 (0)	32,481 (84.4)
**Cephalosporins**	10,757 (14.4)	0 (0)	1,641 (15.3)	0 (0)	9,116 (84.7)
**Macrolides**	10,574 (14.2)	355 (3.4)	0 (0)	1,318 (12.5)	8,901 (84.2)
**Quinolones**	4,693 (6.3)	0 (0)	0 (0)	2,168 (46.2)	2,525 (53.8)
**Lincosamides**	4,732 (6.3)	0 (0)	0 (0)	0 (0)	4,732 (100)
**Nitroimidazoles**	3,687 (4.9)	288 (7.8)	0 (0)	0 (0)	3,399 (92.2)
**Aminoglycosides**	1,743 (2.3)	0 (0)	0 (0)	198 (11.4)	1,545 (88.6)
**Total**	**74,648**	**6,297 (8.4)**	**1,641 (2.2)**	**3,684 (4.9)**	**63,026 (84.5)**

Table 3 compares the distribution of appropriateness of antibiotics use by physicians’ and patients’ characteristics. In the second column, “combined use” refers to physicians prescribing more than one group of antibiotic for the same patient in the same visit. The percentage of appropriate antibiotics use was reduced from that shown in Table 1 because two or more antibiotics prescribed to the same patient on the same day was considered as one visit. The percentage of each prescription type was thus appropriate use (8.7%), combined use (7.8%), escalated use of extended spectrum (1.9%), incorrect spectrum (4.3%) and unnecessary use (77.3%).

**Table 3:** Factors associated with inappropriate use of antibiotics on bivariate analysis.

Characteristic	Appropriate use	Inappropriate use, n (%)				Crude OR (95% CI)	Total N (%)
	Frequency (%)	Combined use	Escalated use of extended spectrum	Incorrect spectrum	Unnecessary use		
**Total**	**4,966 (8.7)**	**4,463 (7.8)**	**1,071 (1.9)**	**2,436 (4.3)**	**44,073 (77.3)**		**57,009**
**Physician characteristics**							
**Sex**							
Female	2376 (10.6)	1,565 (7.0)	482 (2.1)	1184 (5.3)	16,821 (75.0)	Ref	22,428 (39.3)
Male	2590 (7.5)	2,898 (8.4)	589 (1.7)	1252 (3.6)	27,252 (78.8)	1.46 (1.38, 1.55)	34,581 (60.7)
**Age group (years)**							
25–31	1925 (8.8)	1,779 (8.1)	506 (2.3)	779 (3.5)	16,995 (77.3)	Ref	21,984 (38.6)
32–38	1345 (7.4)	1,466 (8.1)	353 (2)	660 (3.6)	14,266 (78.9)	1.19 (1.11, 1.29)	18,090 (31.7)
39–75	1696 (10.0)	1,218 (7.2)	212 (1.3)	997 (5.9)	12,812 (75.7)	0.86 (0.8, 0.92)	16,935 (29.7)
**Education**							
College	2082 (9.9)	1,676 (7.9)	564 (2.7)	716 (3.4)	16,064 (76.1)	Ref	21,102 (37.0)
Junior college	2278 (8.5)	1,923 (7.2)	356 (1.3)	1405 (5.2)	20,812 (77.7)	1.18 1.11, 1.25)	26,774 (47.0)
Technical secondary school	606 (6.6)	864 (9.5)	151 (1.7)	315 (3.4)	7,197 (78.8)	1.54 (1.40, 1.69)	9,133 (16.0)
**Work duration (years)**							
≤5	1485 (9.5)	1,373 (8.8)	242 (1.5)	488 (3.1)	12,098 (77.1)	Ref	15,686 (27.5)
6–10	1624 (9.6)	812 (4.8)	533 (3.2)	660 (3.9)	13,285 (78.5)	0.98 (0.91, 1.06)	16,914 (30.0)
11–20	924 (6.3)	1,435 (9.7)	194 (1.3)	606 (4.1)	11615 (78.6)	1.57 (1.44, 1.71)	14,774 (25.9)
21–30	411 (6.7)	547 (9.0)	73 (1.2)	518 (8.5)	4548 (74.6)	1.45 (1.29, 1.62)	6,097 (10.7)
≥31	522 (14.8)	296 (8.4)	29 (0.8)	164 (4.6)	2,527 (71.4)	0.60 (0.54, 0.67)	3,538 (6.2)
**Professional title**							
Resident physician	4,001 (8.7)	3,523 (7.7)	845 (1.8)	1757 (3.8)	35,623 (77.9)	Ref	45,749 (80.2)
Attending physician	830 (9.4)	881 (9.9)	218 (2.5)	584 (6.6)	6,349 (71.6)	0.93 (0.86, 1.00)	8,862 (15.5)
Associate chief physician	135 (5.6)	59 (2.5)	8 (0.3)	95 (4)	2,101 (87.6)	1.61 (1.35, 1.93)	2,398 (4.3)
**Patient characteristics**							
**Sex**							
Female	2,604 (8.5)	2,454 (8.0)	703 (2.3)	1395 (4.5)	23,579 (76.7)	Ref	30,735 (53.9)
Male	2,362 (9.0)	2,009 (7.6)	368 (1.4)	1041 (4)	20,494 (78.0)	0.94 (0.88, 0.99)	26,274 (46.1)
**Age group (years)**							
≤5	947 (10.6)	675 (7.5)	41 (0.5)	416 (4.6)	6,881 (76.8)	Ref	8,960 (15.7)
5–17	1,234 (12.8)	765 (7.9)	88 (0.9)	274 (2.8)	7,271 (75.5)	0.80 0.73, 0.88)	9,632 (16.8)
18–49	1,472 (7.7)	1,222 (6.4)	501 (2.6)	916 (4.8)	14,962 (78.4)	1.41 (1.30, 1.54)	19,073 (33.5)
50–64	706 (6.5)	966 (8.9)	263 (2.4)	473 (4.4)	8,404 (77.7)	1.69 (1.53, 1.88)	10,812 (19.0)
≥65	607 (7.1)	835 (9.8)	178 (2.1)	357 (4.2)	6,555 (76.8)	1.54 (1.39 1.72)	8,532 (15.0)
**Antibiotic route**							
Injection	911 (5.6)	1,796 (11.0)	749 (4.6)	393 (2.4)	12,492 (76.4)	Ref	16,341 (28.7)
Oral	4,055 (10.0)	2,667 (6.6)	322 (0.8)	2043 (5)	31,581 (77.7)	0.53 (0.49, 0.57)	40,668 (71.3)
**Payment source**							
Out-of-pocket	614 (10.2)	487 (8.1)	132 (2.2)	404 (6.7)	4,410 (72.9)	Ref	6,047 (10.6)
New rural cooperative- medical care system	4,352 (8.5)	3,976 (7.8)	939 (1.8)	2032 (4)	39,663 (77.8)	1.21 (1.11, 1.32)	50,962 (89.4)

OR: Odds ratio, CI: Confidence interval, Ref: Reference group

The crude odds ratios indicate the strengths of the association between inappropriate use and physicians’ and patients’ characteristics. Male physicians were more likely to prescribe antibiotics inappropriately as were those aged 32–38 years (compared to those aged less than 32 years), those who had not completed a college degree, worked for 11–30 years (compared to those who had worked for less than 5 years), and were associate chief physicians (compared to resident physicians). Patients who were female, aged 18 years or more (compared to those aged ≤5 years), received antibiotics intravenously and received financial assistance from an insurance scheme were more likely to be prescribed antibiotics inappropriately.

Table 4 shows factors associated with inappropriate antibiotic use on multivariate analysis. On the physician's side, being male, aged less than 32 years, having a lower level of education, and being an associate chief physician were associated with a higher likelihood of inappropriate antibiotic use. Increasing work duration did not equate to a higher odds of inappropriate antibiotic use. However, compared to newly employed physicians, those who had a work experience duration of more than 5 years were more likely to prescribe antibiotics inappropriately. Antibiotics prescribed intravenously were more likely to be inappropriate. On the patient's side, younger patients (age≤17 years) were more likely to get appropriate prescription. Finally, patients who received financial assistance from an insurance scheme were more likely to be prescribed antibiotics inappropriately.

**Table 4:** Factors predicting inappropriate use of antibiotics on multivariate analysis.

Characteristic	Adjusted OR (95% CI)	P value
**Physicians** **Sex: ref = Female**		
Male	1.65 (1.54, 1.78)	< 0.001
**Age: ref = 25–31 years (years)**		
32–38	0.58 (0.53, 0.64)	< 0.001
39–75	0.30 (0.27, 0.35)	< 0.001
**Education: ref = College**		
Junior college	1.30 (1.21, 1.39)	< 0.001
Technical secondary school	1.60 (1.42, 1.80)	< 0.001
**Work duration: ref = ≤ 5 (years)**		
6–10	1.36 (1.25, 1.48)	< 0.001
11–20	3.14 (2.78, 3.54)	< 0.001
21–30	3.80 (3.20, 4.53)	< 0.001
≥ 31	1.54 (1.29, 1.83)	< 0.001
**Professional title: ref = Resident physician**		
Attending physician	0.97 (0.87, 1.08)	0.60
Associate chief physician	1.99 (1.62, 2.43)	< 0.001
**Patient** **Sex: ref = Female**		
Male	0.98 (0.92, 1.04)	0.46
**Age: ref. < 5 (years)**		
5–17	0.76 (0.69, 0.83)	< 0.001
18–49	1.25 (1.14, 1.36)	< 0.001
50–64	1.36 (1.22, 1.50)	< 0.001
≥ 65	1.20 (1.08, 1.34)	< 0.001
**Route: ref = Injection**		
Oral	0.49 (0.45, 0.53)	< 0.001
**Payment source: ref = Out-of-pocket**		
New rural cooperative medical care system	1.18 (1.08, 1.30)	< 0.001

OR: Odds ratio, CI: Confidence interval

## Discussion

This study showed five patterns of antibiotic use, namely: 1) appropriate use, 2) unnecessary use, 3) escalated use of extended spectrum antibiotics, 4) incorrect spectrum of antibiotics, and 5) combined antibiotic use. More than 90% of antibiotic prescriptions were inappropriately prescribed. Respiratory infection was the most common disease linked with antibiotic prescriptions. While penicillins were the most commonly prescribed antibiotic, cephalosporins and macrolides were more inappropriately prescribed, particularly due to escalation and incorrect spectrum of antibiotic. These antibiotics could have been de-escalated to penicillins. The background education of these primary care physicians was generally below college level. A physician's low level of education and senior position were significantly associated with inappropriate antibiotic prescription. Antibiotic misuse was also associated with injection route as well as adult patients and those who had insurance.

In general, uncomplicated respiratory infections are mostly caused by viruses, which cannot be treated with antibiotics [15]. Unnecessary use of antibiotics to treat such infections has been reported in many countries such as Italy (67%), Norway (11%), USA (42%), Qatari (45%), Canada (84%) and China (71%) [10, 16–20]. In our study, unnecessary antibiotic use accounted for 77% of the prescriptions. Misuse and overuse of antibiotics could increase the duration of the disease and associated costs and increase resistance to infections as well as increase the risk of adverse drug reactions [21]. Therefore, it is important to identify antibiotic patterns as they vary by different factors such as the medical education system of a country.

As much as 29% of antibiotics used in this study was administered by injection. Overuse of injections was also seen other countries such as Vietnam, India, and Korea [22–24]. This practice is rarely needed in primary care as most infections can be controlled with oral antibiotics [25]. Injection of drugs is complicated by serious adverse drug reaction and diseases complications such as local infections, bleeding, and nerve injury [24, 26]. There are many guidelines and punitive measures in China that guide physicians on how to use antibiotics, but there are no such guidelines as how physicians should talk to patients and families about whether antibiotics are needed or not, and discuss possible harms [21].

Most of the antibiotic prescriptions in this study were made by resident physicians with a below college level of education, and this was associated with antibiotic overuse and misuse. Based on this evidence, refresher courses on antibiotic prescribing for primary care physicians are necessary [7, 9]. The training should emphasize avoidance of incorrect and unnecessary use, narrow-spectrum antibiotics use, and when to prescribe injection antibiotics.

Furthermore, there are no measures to teach patients how to use antibiotics and manage symptoms of non-bacterial infections. Patient’s attitude, education level and expectations play a key role in inappropriate antibiotic prescribing by physicians in outpatient settings. Patients who are misled by the efficacy of antibiotics were prescribed antibiotics more frequently than those who were not expecting them [27]. Patients need to be given more information concerning antibiotics use. Easy-to-understand health education materials can help empower patients and consequently change inappropriate antibiotic prescribing practices by physicians [27, 28]. Studies from Italy and the UK have shown that the Internet and social media can be an effective way for relevant government departments and medical institutions to disseminate the right principles of antibiotic use to patients [29, 30].

Our study has several limitations. Firstly, the results of our study cannot be generalized to hospitals with fewer outpatient physicians and to hospitals outside of our study setting. Secondly, the whole analysis of this study was based on data keyed in by the primary physicians. While the antibiotic information is very accurate, that on disease classification might be less so. Accurate diagnosis is a prerequisite of appropriate selection of antibiotic. Data on appropriateness of antibiotic choice in this study must therefore be interpreted with caution. Thirdly, the time frame of the survey is limited, diseases and medications in different seasons will vary, which is not covered in this study.

## Conclusion

Overuse of antibiotics for uncomplicated respiratory infections, use of cephalosporins, macrolides and injection antibiotics in primary care are the major problems of clinical practice in the study areas.

## Supporting information

S1 File Summary of National Health Commission of China for Guiding Principle of Clinical Use of Antibiotics introduced in 2015 related to this study. (DOCX)

S1 Table Database of antibiotic prescriptions. (XLSX)
