# Supplementary material for: Clinical pattern of antibiotic overuse and misuse in primary healthcare hospitals in the southwest of China
Source: PLoS One. 2019 Jun 26;14(6):e0214779. doi: 10.1371/journal.pone.0214779 (PMC6594576; doi:10.1371/journal.pone.0214779)
Supplement: S1 File — (DOCX) [file pone.0214779.s001.docx]

**S1 File: Summary of National Health Commission of China for Guiding Principle of Clinical Use of Antibiotics introduced in 2015 related to this study**

1. **The diagnosis of bacterial infection has an indication for the application of antibiotics.**

According to the patient's signs and symptoms, laboratory tests and image examination results are required to determine the indications for application of antibacterial drugs.

1. **Cases without pathogenic results can be taken empirical therapy according to the clinical manifestation.**

For patients clinically diagnosed with bacterial infection, the possible pathogens can be inferred from the infection site, the underlying disease, the incidence, the place of onset, the history of previous antimicrobial drugs and their therapeutic response, and combined with local bacterial resistance monitoring data, in the case of unknown etiological results. For patients with negative culture results, further diagnostic measures can be taken according to the effect of empirical treatment and the patient's situation.

1. **The antimicrobial therapeutic regimen is developed by synthesizing the patient's condition, pathogenic bacteria and antimicrobial characteristics as follows:**

- Highly targeted, narrow-spectrum, safe and affordable antimicrobial drugs should be given priority, according to possible pathogens and local drug resistance status.
- For the majority of patients with mild to moderate infections, oral treatment should be given first, and oral and well-absorbed antimicrobial varieties should be selected. Local application of antibiotic drugs should be avoided as far as possible.
- The duration of antibiotic use depends on changes in systemic and local symptoms. It is usually stopped 72 to 96 hours after the body temperature returns to normal, or after the symptoms of local infection disappear completely.
- The combination of antibiotic drugs is not required, except in cases of severe mixed infections, immune defects, drug-resistant bacterial infections that require prolonged treatment, and the use of more toxic antibiotics.

**Appendix: Principles of empirical antibacterial treatment for various bacterial infections**

| **Disease classification** | **Common pathogenic bacteria** | **Main diagnosis and treatment principles** | **Preferred antibiotic** |
| --- | --- | --- | --- |
| Acute bacterial pharyngitis and tonsillitis | Group A hemolytic Streptococcus | The pathogen was diagnosed by pharyngeal swab culture and rapid antigen test (RADT) before administration, and antimicrobial drugs were selected for hemolytic streptococcus infection. | Intramuscular injection of penicillin G, oral amoxicillin |
| Acute bacterial sinusitis | Pneumonia chain Haemophilus influenzae | Initial treatment should cover Streptococcus pneumoniae, Haemophilus influenzae and Mocha, such as amoxicillin/clavulic acid, and then the drug should be adjusted according to the treatment reaction, bacterial culture and drug sensitivity test results. | Oral amoxicillin |
| Acute tracheal-bronchitis | Most are viral infections, and a few are mycoplasma, chlamydia or pertussis Bordetella infections. | Symptomatic treatment is the mainstay and antibiotics should not be routinely used. | If a few are mycoplasma, chlamydia or pertussis Bordetella infection, macrolides are preferred. |
| Periodontitis, pericoronitis, around the apex | Streptococcus mutans | Local treatment is the main, antimicrobial treatment as a supplement. | Oral amoxicillin |
| Acute cholecystitis | Escherichia coli, enterococcus | Consider cholecystectomy and antimicrobial therapy, as appropriate. | Penicillin and second-generation cefuroxime vein Administration |
| Vaginitis | Trichomonas, candida albicans | For vaginal secretion smear and culture. Choose drugs based on pathogens and the absence of a combined infection. | Local medication or oral of metronidazole and mycin |
| Suppurative cervicitis | Neisseria gonorrhoeae, chlamydia trachomatis | For culture or nucleic acid testing, the dosage and course of the antimicrobial drug must be sufficient. | Gonococcal Cervicitis with three generations of cephalosporins, non-gonococcal cervicitis oral polycyclic cyclosporine, azithromycin |
| Pelvic inflammatory | Neisseria gonorrhoeae, enterobacter bacteria | Secretion and pelvic pus and other specimens to do pathogenic testing, the emergence of mixed infection, can use broad-spectrum antibiotics. | Second or third generation cephalosporins + metronidazole / tinidazole + doxycycline / azithromycin |
| Bone, joint infection | Staphylococcus aureus | Choose antimicrobial drugs with high concentration of drugs in bone and joint cavity and not easy to produce drug resistance. | Oxacillin, cloxacillin, Amo Xilin/Clavulanic acid, ampicillin/ Shubatan |
| Bacterial conjunctivitis | Haemophilus influenzae, streptococcus pneumoniae, staphylococcus aureus, neisseria gonorrhoeae | Mainly for local application of antibiotics, accompanied by pharyngitis or acute suppurative otitis media, or infected with Haemophilus influenzae, should be oral antimicrobial drugs at the same time. | Ofloxacin/erythromycin topical or oral |
| Acute cellulitis | A Group of hemolytic streptococcus | A systemic, severe or complex skin and soft tissue infection that requires the whole body to be used as an antimicrobial drug. | Penicillin, amoxicillin oral |
| Rheumatic fever | Streptococcus | Eliminates the remaining streptococcus infection lesions. | Penicillin oral |
